# Collectanea

**Published:** 1826-12

**Authors:** 


### COLLECTANEA.

Floriferis ut apes in saltibus omnia libant,

Omnia nos, itidem, depascimur aurea dicta.

### PHYSIOLOGY.

*Effects of Hyoscyamine and Atropia.*—Dr. F. Reisinger, of Landshut, has performed some experiments with a view to ascertain the effects of Hyoscyamine and Atropia, an account of which is given in the Edinburgh Medical and Surgical Journal, from the Med. et Chir. Zeitung. The Doctor says—

Hyoscyamine, prepared from the henbane seed, I found to be an extremely-powerful substance for the dilatation of the pupil. A small drop of a solution of hyoscyamine (gr. 1 to ℈ss. of water) was introduced into the eyes of some dogs and cats; the eye was scarcely at all irritated in any case, and the pupil was so considerably widened, that, an hour after the application of the solution, only a small ring of the iris could be seen beyond the edge of the cornea; and, after three hours, the pupil appeared as large as the cornea itself, without the power of vision being diminished, or any other bad symptoms being induced, even when the solution was introduced into both eyes. After three days, the dilatation of the pupil first began to diminish, and it was not before the sixth day that the iris recovered its natural state. A drop of a solution of extract of hyoscyamus, containing five grains of the extract to half a scruple of water, produced in the same eyes a considerable irritation for the space of from five to eight minutes, which was shown by the secretion of tears, shutting of the eye-lids, rubbing of the eye-brows with the feet, &c.; and a much less complete dilatation of the pupil, which in dogs disappeared after six or eight, and in cats after twenty-four hours. As soon as we learnt by these experiments, which we frequently repeated, that the hyoscyamine did not, in its action, injure either the conjunctiva, or any of the deeper-seated organs, as, for instance, the retina, I proceeded to apply it on the human eye, and found that a drop of a solution of one grain of hyoscyamine in a drachm of distilled water, applied to the eye of a cataract patient 71 years old, produced such a dilatation of the pupil, that only a small ring of the iris was apparent. The pupil continued dilated seven days, during which time the old woman could see moderately well, and no irritation whatever was produced in any part of the eye. At another time, a drop of a solution of five grains of extract of hyoscyamus, in half a scruple of water, applied to the same eye at another time, produced a considerable burning, and only a moderate dilatation of the pupil after twelve hours. From other experiments with the hyoscyamine, we obtained nearly the same results. The hyoscyamine, which was obtained from the stalk and leaves of the plant, irritated the eye much more, and was less efficacious than that obtained from the root.

The experiments made with the atropia, under nearly the same circumstances, produced results similar to those obtained from the hyoscyamine. The atropia prepared from the root scarcely appeared to excel that obtained from the other parts of the plant in its efficacy. Both kinds of atropia acted on the eyes of animals very powerfully, so that the iris was almost invisible; but this extreme dilatation did not continue more than two days. Although it may be necessary to make a greater number of experiments to determine which of the two substances is the most active, still there can be no doubt of the superior excellence of the narcotic bases of these plants over the extracts which are generally used. In iritis, and preparatory to the different operations for cataract, they certainly deserve to be used in preference.

Dr. Kopp recommended the extract of hyoscyamus to be introduced into the rectum previous to the operation for fistula in ano, for the purpose of dilating the gut; he also advised the extract to be used in the form of clysters, for allaying spasmodic contractions of the rectum, and in that of ointment to be applied to the mouth of the uterus for the purpose of facilitating difficult births. In all these cases, and in several others, I would suggest the substitution of the hyoscyamine and the atropia for the extracts. At any rate, these substances ought to be used under these circumstances in preference to opium, which acts with so much irritation on the mucous membranes. Whether the internal use of these new medicines be attended with such advantage remains to be determined by the result of new and careful experiments; still, judging from analogy, I think that, in spasmodic affections, their internal use is preferable to that of opium.

My own investigations into the subject are very limited, and confined to the following observations. A dog, three months old, swallowed, at ten in the morning, a grain of atropia, obtained from the root of the plant. In half an hour, the ears and tail began to hang, and the dog became inactive, surly, and sleepy. The pupils also were moderately dilated. Towards twelve the dog began to recover itself, and showed a disposition for his accustomed food; but, at one, the animal became very uneasy, moaned frequently, lay flat on the ground, and slept at intervals. Weakness, and particularly loss of muscular power, were very apparent; the power of vision was also gone, as the dog tumbled over every thing. As the whole of the body was flaccid, and not in the slightest degree tender, I supposed that the moaning was not produced by pain, but by the loss of his sight, and fear of light. At two, I took care that he should take some water and vinegar, with plenty of butter. Still the animal continued in the same state; it also now occasionally yawned; the nose was quite dry; the pupil still more dilated; and the palpitation of the heart was strong and rapid. The passage of the urine and feces was natural. At half past three vomiting of the undigested food took place; at five the dog recovered itself considerably; and at seven he was quite restored. Another dog, just of the same age, swallowed at the same time a grain of extract of belladonna. In half an hour's time, the same symptoms came on as in the other, but in three hours he was perfectly recovered. The same dose of atropia and hyoscyamine were given to some half-grown rabbits, but it did not produce the least effect on them. These animals appear to be very insensible both to the external and internal action of the substances. It is extremely curious, that, as soon as the slightest particle of these narcotic bodies was introduced into the mouth of a cat, a copious flow of saliva and of foam came on, and at the same time the animal began to chew and loll out the tongue. I conclude these remarks with a wish, that those medical men who have it in their power will give these principles a trial, and communicate the results of their observations to the public.

### *On the Structure of the Eyes of Insects.* By Mr. William Ewing, of Glasgow.

*Simple Eyes.*—These are the eyes with which we find insects provided in the first state of their existence, as well as those that are produced perfect from the egg. They differ, indeed, as to number, situation, and arrangement, in those insects, but they are identically the same: their structure is that of a double convex lens, but more convex without than they are within; they are all transparent when cleaned, and capable of refracting light: they are of a hard substance, and change not on being taken from the insect.

*Conglomerate Eyes.*—They are exactly the same as simple eyes, being double convex lenses, each of them capable of refracting light: only they are more numerous than simple eyes, and are collected into patches, and have a common retina.

*Compound Eyes.*—Under this head are included the whole of those eyes which we find provided with a lace-like covering. I am satisfied, however, that, under this division of the subject, there are different modifications, which I shall notice separately.

*Beetle's Eyes.*—It appears to me that those eyes differ not from simple eyes of the conglomerate kind. The form indeed varies, but the structure is the same. It is spherical, and composed of a number of hexagonal apertures, filled with lenses, each of which possesses the same properties as simple eyes; and having a common retina, which is connected to the external covering, so as to exhibit the appearance that two watch-glasses would have if they were cemented together.

*Butterfly's Eye.*—This eye differs from all the insects' eyes I have examined. It consists of a ball which is orbicular, and of a dark purple colour towards the external lace-like covering; and on the other side, where the optic nerve enters, it is white and less convex. This ball occupies a circular cavity formed by the external covering and the retina, and is surrounded by a very clear gummy liquor, into which it appears to move. My way of ascertaining this was by fixing the live insect in the pliers under the microscope, and, putting a mark on the centre of the external covering, I turned the insect backwards and forwards; and I observed that, when the lace-like covering moved round, the dark spots in the eye were stationary, and could be moved from one edge of the eye to the other. Now this may either be a reflection, or it may be the dark orbicular side of the ball above mentioned, shining through the limpid gummy liquor into which it floats. There are no lenses in the lace-like covering of the eyes of this insect, but they are lined with a thin transparent membrane betwixt the external covering and coating of the eye.

The eye of the night-butterfly, or moth, has the same structure as the one just described, only it is dark when viewed in the light; but, if examined in the shade, it shines with a beautiful yellowish lustre. This is emitted from the ball of the eye after being extracted from the insect. There is a peculiarity in the eye of this insect, which I cannot find in any other I have examined: viz.—from the hexagons in the external covering proceed tubes, which convey the apertures through the dark coating of the eye. They are smallest next the ball, are hard and transparent, and appear to be of the same substance as the external covering. There are no lenses in the covering of the eye of this insect.

The next modification of compound eyes belongs to a very numerous class of insects of the fly kind, (and it is to this class chiefly I think the term can be applied:) viz. to all those which are provided with stemmata, (a kind of eyes which I shall next mention.) As I have not been able to discover the effect of these as organs of sight, I shall merely state, that, in all those appendages called compound eyes in insects having stemmata, I could never find lenses, nor any internal organisation similar to those that have them.

*Stemmata.*—These are eyes with which the greater number of bees and flies are provided, and they appear to be their real eyes. They are exactly similar to, and capable of the same properties as simple eyes: they are variously situated in various insects, but in all of them which I have tried, if they are shut up, the insect is rendered blind.

In the foregoing remarks, I have merely mentioned the result of many experiments, from which I have preserved specimens of various eyes, which prove the facts stated; and since the oculi of insects arrange themselves under three different modifications, I have suggested the three following queries, which I hope some of your learned correspondents will be able to solve:—

Query—How is vision performed in those insects of the butterfly kind, since they are not provided with stemmata, nor have lenses in the hexagonal apertures, in the external covering of their eyes?

Query—How is vision performed in those insects which we find unprovided with stemmata, but in the external covering of whose eyes we find the hexagonal apertures filled with double convex lenses?

Query—How is vision performed in those insects which we find provided with stemmata, but want lenses in their compound eyes?

(*Brewster's Edinburgh Journal of Science.*)

---

### PATHOLOGY.

*Perforations of the Œsophagus and Stomach.*—M. Leveillé read two cases of this kind: one was in a man, twenty-two years of age, who, six weeks before he was taken ill, had been bitten on the fingers of the right hand by a dog, which was immediately killed, without ascertaining whether it was rabid or not. The wound was quickly healed; but, six weeks after, suddenly very sharp pains came on in the right shoulder and part of the chest of the same side; a feeling of strangulation, rendering the act of swallowing very difficult, accompanied these. A large bleeding did not afford any relief. Delirium soon came on, with great agitation; the patient uttered frightful cries; the slightest touch produced the effect of an electric shock; he drank with avidity; vomited a glutinous yellow matter several times in the evening; and died in the night. On opening the body, the œsophagus, about an inch and a half above its passage through the diaphragm, had a perforation of seven or eight lines in length, the edges of which were smooth and thin, and through which an effusion had taken place into the posterior mediastinum. M. L. thinks this perforation was caused by a pustule, which, developing itself in the coats of the œsophagus, had ulcerated through them in one point.

The second case is that of a man of fifty-five years of age, who, having always enjoyed good health, experienced in the course of one year four fainting fits, which lasted only a few minutes; and for which, sinapisms to the feet, leeches to the anus, and purgatives, were used. There soon appeared symptoms of disease of the stomach, with pain after eating, want of appetite, loss of much blood per anum, prostration of strength, and wasting of the flesh. The patient was put on a milk diet. Soon after digestion invariably excited pains in the stomach. There was no swelling to be felt in the epigastric region, and there was no vomiting, but obstinate constipation. One evening, the patient suddenly experienced great pain in the hypochondrium, which increased during the night; the belly became swollen and tense; and he died in the morning. On opening the body, there was found, on the anterior surface of the stomach, near its small curvature, four fingers' breadth from the pylorus, an oval opening, the circumference of which was from eight to ten lines; but the mucous membrane was completely destroyed to a much greater extent, and from the margins of the hole several yellowish tubercles, as large as a grain of barley, projected. The stomach in other respects was sound. The left auricle of the heart had double the usual capacity; and, on the partition between the auricles, there was a tumor of an encephaloid appearance, forty-five lines in circumference, and which probably had been the cause of the syncope.

M. L. remarks that, in both these cases, it was impossible for the most acute physician to form a diagnosis, or to predict the seat or nature of the disease. (*Archives Generales.*)

---

### SURGERY.

*Removal of the Greater Portion of the Lower Jaw-bone.*—Mr. Lizars, of Edinburgh, has recently performed this operation in a case of osteo-sarcoma. We subjoin the particulars, as related by him in the Edinburgh Medical and Surgical Journal:—

Upon careful examination of the tumour, I found it of the size of a very large orange, having a firm adhesion to the right side of the inferior maxillary bone, and extending downwards and outwards, or sacrad and peripherad, and locking the inferior to the superior maxillary bone, in consequence of its magnitude and fixed position; so that the mouth could not be opened to examine the extent of the tumour internally or mesiad. By the use of a mirror, however, I could perceive that it extended nearly two inches from the lower jaw-bone towards the body of the tongue; and on inserting the finger between the cheek and the bone, the tumour felt hard, and appeared either cartilaginous or osseous, and extended from the symphisis menti back to the angle, and occupied even a little of the ramus. The tumour felt also hard along the base of the bone, and around all its own base, but from this, outwards to its apex, became progressively softer. After careful examination and due deliberation, I replied to Dr. Scott, that the tumour appeared to be osteo-sarcoma of the lower jaw-bone, and that nothing but the entire removal of the diseased bone would eradicate the disease, and give the patient a chance of life; for in all the cases I had witnessed, where the tumour had been hammered or chiseled off the bone, the affection had returned, or rather the patient lingered out a most loathsome existence, from malignant ulceration and hectic fever.

Wednesday, the 16th of August, was appointed for the operation, on which day Dr. Scott did me the favour to be present; and, on the Monday preceding, I requested the opinion of my able friend Dr. Poole, respecting the propriety of at once removing the bone, who judiciously observed, that it was my duty to ascertain, during the operation, whether the bone was affected or not. On Tuesday the patient took a brisk purge, which operated well.

The patient being laid on a mattress placed on a table, with his head towards the window, on a pillow, I made an incision through the skin from the mastoid process of the temporal bone around the inferior or sacral aspect of the tumour, embracing at the time every portion of diseased skin, onwards to the symphisis menti. In the region of the sterno-cleido-mastoideus muscle, I made a second cutaneous incision from near the middle of the preceding, downwards or sacrad, parallel with that muscle, to the extent of two inches, in order that I might be able to insulate the tumour with more freedom. I then made a third incision from where the first began, in the region of the mastoid process of the temporal bone, across the ramus of the lower jaw-bone, and along its body to the symphisis menti, onwards for an inch to the opposite or left side. I made this last incision at once into the mouth, whenever I had got beyond the masseter muscle, and immediately made an incision along the lower jaw-bone into the substance of the tumour; and requested Drs. Duncan, Poole, and Borthwick, who were present, to examine the condition of the bone, which, on inspection, they considered healthy. I therefore proceeded to insulate the tumour, by dissecting back the integuments from its posterior aspect, holding the edge of the knife towards the tumour and trachea; but on deepening at the angle of the bone, I found the tumour formed by the bone, and perfectly incapable of being detached from it. In my attempts at this moment, the tumour burst, and discharged a glairy honey-like fluid, apparently of the mellicerous nature, and I therefore removed the loose portion of it, by cutting towards the trachea. In this stage of the operation, a multiplicity of arteries sprang, and required to be secured,—particularly towards the angle of the bone,—and were extremely embarrassing, and difficult to be seized hold of. I now resolved on removing the bone; and proceeded by rendering it rudely clean at the symphisis, where I found the disease extended so far to the left side as to involve the four incisive teeth; I attempted to snap the bone at once with Mr. Liston's forceps, but was compelled first to use a small saw, in order to weaken it; I next did the same across the middle of the ramus, using first the saw and then the forceps, and cautiously avoiding the internal maxillary artery. I then requested my assistant, Mr. M'Crae, a very intelligent young surgeon, to elevate the bone gently at the angle, to enable me to detach it, together with what remained of the tumour, by sweeping with the scalpel, having its cutting edge towards the chin and trachea, which was soon accomplished. The lingual artery, with some others, sprang at this period; but the bone being removed, and there being an open space, they were easily secured. I shall never forget the able assistance which I received from Dr. Poole at this period. I now carefully examined every point of surface of this extensive wound, and removed, I trust, every vestige of disease. The long ends of all the ligatures being cut off, and the wound cleanly sponged, the lips were approximated as far as they would admit with thick strong ligatures, and compresses of lint, with a four-headed roller, applied. The patient was put to bed, and a draught with forty-five drops of laudanum given.

Before proceeding with the remaining detail of this interesting case, I shall make a few observations on the operation. Had the other gentlemen been as convinced as I was myself that the bone was diseased, and had I proceeded as follows, which was my original intention, the operation would have been one of the simplest in surgery; but, from the division of it into two stages, first, the attempt to remove the tumour, and save the bone, and, secondly, the ultimate removal of the bone, it was rendered one of the most complex I ever performed. I have more than once been sadly perplexed in removing tumours at the angle of the lower jaw-bone, in consequence of this bone overhanging the tumour, and obscuring the arteries; and it appears to me a subject of consideration for the operator, when large tumours are situated at the angle of the inferior maxillary bone, and burrow beneath it towards the fauces and tongue, either to secure, in the first place, the common carotid artery, or to remove a portion of the bone. In this patient, had the tumour been merely adherent to the bone, and the latter not formed a portion of the former, it would have been next to an impracticability to remove all the diseased structure without wounding more blood-vessels. I felt myself quite paralysed in removing the disease mesiad of the angle of the bone, as every incision wounded an artery, and it was scarcely possible to secure them from their depth. My intention in this operation was to remove the bone, together with the tumour, as I have described in page 45 of Part 9 of the Anatomical Plates; to proceed immediately after the upper incision had exposed the lower jaw-bone, to notch it across at the symphysis and ramus, and then to remove the bone and tumour, by cutting from the sterno-cleido-mastoideus muscle towards the apex of the tongue and trachea. In this way the operation would ha[illegible] been safe, rapid, and easy of accomplishment. In all such operations, the cutting edge of the knife should be held towards the trachea, and the tumour dissected from the region of the carotid towards the trachea, and not, as Langenbeck recommends, from the trachea towards the carotid. In the latter case, the operator may require to secure the same artery more than once, and runs a greater risk of wounding the carotid artery and internal jugular vein; he also cannot repress, with the fingers of his left hand, these important blood-vessels so easily and so securely. In this operation, when I divided the cheek, the facial artery did not bleed, having been obliterated by its passage through the tumour, but the transversalis faciei required a ligature.

*Appearances of the Tumour*—The conical portion, which was first removed, has a thick closely compacted cyst immediately beneath the integuments: centrad or interior to this, is a thick cartilaginous stratum; and still more centrad or interior, is a granulated surface, with some long spiculæ of cartilage,, forming the parietes of the sac or cyst in which the mellicerous fluid was contained.

The bone, comprehending the four incisive, together with the canine and molar teeth of the one side, has cartilaginous projections where the tumour adhered, and distinct exostoses near the symphisis on the outer or dermal aspect, and on the inner or mesial aspect of the angle; and at all these points it cuts soft like cartilage, and the bone itself is involved in the same softened mass.

The patient did well; and the last report (dated September 2,) runs as follows:—

The wound has been daily dressed with the adhesive straps, and the flaps forming the aperture leading into the mouth can now be approximated. The gape is exceedingly small, being about the twelfth of an inch in breadth, and only an inch in length. He feels no pain in any part of the wound, and both the cut ends of the bone are covered with granulations, which have cicatrized. He can swallow any pulpy mess, as porridge, soups, broths, and minced meat; he sits out of bed, and walks about his room, and is now regular in his bowels.

*Removal of Calculi from the Bladder.*—Mr. G. Bell, in a paper in the Edinburgh Journal of Medical Science, gives the following account of an ingenious method practised by a gentleman on his own person:—

I shall only farther slightly allude to a very simple instrument, contrived by a Scottish clergyman for his own use. He had laboured for many years under enlarged and indurated prostate: symptoms of stone supervened; perpetual retention of urine, which was never passed for years without the catheter, by the eye of which several small calculi were at different times brought out. His urethra was of very large caliber, quite callous, bearing any freedom. He cut off the vesical extremity of the catheter, had it plugged up very neatly with a ball of silver, attached to a silver wire, to enable it to pass smoothly into the bladder. When introduced, the ball was withdrawn; his forefinger applied to the outer orifice of the tube, and, after discovering the quarry of stones, he removed his finger, when the sudden gush of urine in a large stream often brought one or two calculi along with it. When I saw him in Galloway, three years since, he had removed upwards of 150 in this way, and he has continued to remove many more since that time; and although it is known, and he is fully aware that he still has at least one very large stone, yet he has experienced so much relief from incessant pains at the neck of the bladder, by having removed so many small calculi, that he cannot bring himself to submit to the removal of the larger calculi by the lateral operation.

*Ectropium of the external Angle of the Eye, cured by a new Operation.* By Professor Walther.—The mode of cure adopted in this case differed essentially from that introduced (we believe) by Sir William Adams, although in each a portion of the tarsus is removed.

An hussar officer, a healthy young man, was wounded near the temple of the left side. The wound shortly healed; but a portion of skin, and of the orbicular muscle of the eye, was lost; there remained also a projecting and unequal scar. The external angle of the eye was drawn towards the temple, about six lines from its natural position; its angular form was lost, and it had now a roundish shape. When the patient applied to Professor Walther, the deformity was considerable, and he experienced also a painful sense of tightness and extension in the temple. He could not perfectly close the eyelids: when he attempted to do so, the middle and internal angle of the lids were brought into a state of apposition, but from the middle to the external angle a division remained. The free use of the eye was also interrupted.

It had been proposed by other surgeons to divide the cicatrix, probably with the expectation of relieving the contraction, which had previously occurred during the healing of the wound. This plan was opposed by Walther: he proposed to unite the eyelids together, beginning at a certain distance from the external angle, so as to form a new angle in the natural position. It was a question, however, how far the growth of the eye-lashes subsequently to the operation, even if they were removed before it, might prevent the union of the parts. It was to be feared, also, that some obstruction to the healing of the parts might arise from the secretion from the Meibomian glands, when the openings of their excretory ducts were removed. The day before the operation, the cilia of both eyelids were removed, by means of a pair of tweezers, as far as it was intended to attempt to unite the parts. The operation was thus performed:—

The tarsus of the under eyelid was drawn out by a pair of forceps, and about one-third of its length removed by a steady sweep of a probe pointed bistoury with a small blade; the incision being extended about one line beyond the external angle. The same portion of the upper eyelid was removed in a similar manner. The two incisions terminated in an acute angle towards the temple. The hemorrhage was trifling. The parts were brought together by two ligatures, which were introduced through the entire substance of the upper and under eyelids, at the distance of about one line from the incised margin. Between the ligatures, which were applied near the two extremities of the wound, the parts were closed by a strip of adhesive plaster. The edges of the wound corresponded accurately at every point, and the opening and shutting of the eyelids did not disturb the parts. Considerable inflammation took place on the second day. The eyelids were much swollen, and the conjunctiva was also inflamed. The inflammation was reduced by bleeding and appropriate treatment. On the fifth day, suppuration had taken place where the ligatures had been introduced, and they were removed. From this time the pain and swelling disappeared; the wound was perfectly united throughout its whole extent; the deformity was entirely removed. The external angle of the eye was now in its natural situation, and the division between the eyelids corresponded on both sides. The patient could move the eyelids freely, and the cicatrix was not perceptible.

In this operation the excretory ducts of the Meibomian glands, or at least their openings, must have been removed, and inclosed within the cicatrix when the parts healed.

It is suggested that the inflammation which occurred might put a stop to the secretion from those glands. Walther concludes that, at all events, this case proves that the commonly received doctrine respecting Hordeolum and Chalazion is incorrect. If these diseases arise from a stoppage of the mouths of the excretory ducts of the Meibomian glands, in this patient there would have been a row of such swellings along the margins of the divided lids.

The non-appearance of the cilia is attributed to the removal of the openings in the skin through which they shoot forth; the bulbs of the hairs being behind the minute apertures within the cicatrix. It appears therefore that, for the growth of the hair as well as plants, the access of the air is necessary. Perhaps, says Professor Walther, from these facts some new plan of cure may be suggested for Dystichiasis and Trichiasis. It would be required to make use of some application which would stop up the mouths of the canals through which the cilia grow so effectually as to exclude the air, by which the subsequent growth of the eyelashes would be prevented, which not unfrequently happens when they have been plucked out for the cure of those troublesome and frequently unmanageable affections.

Professor Walther has lately performed the above operation twice. (*Journal der Chirurgie und Augen-Heilkunde*, band. 9, heft. 1, b. 87.)

*An Improved Mode of Operating for the Piles.* By Dr. J. C. Rousseau.—In this Essay I propose to offer some improvements on the methods adopted for the removal of the piles by a surgical operation; the success of which has been invariable, and warranted by an experience of upwards of twenty years.

When the piles have, from long duration, as well as from a multiplicity of causes, become permanent, and have given rise to fungous excrescences around the anus, not unfrequently attended with ulcerations, hemorrhage, and fistulas, no other alternative is left to relieve the sufferer than the entire removal of the diseased tumors.

Early in my practice, I was so fortunate as to meet with several opportunities that gave me the strongest reasons to deprecate the use of the knife in a number of diseases, but more particularly in the extirpation of piles. Hemorrhages, ulcerations, loss of the substance so necessary around the anus, and the destruction or disability of the sphincter ani, were so frequently observed, that I formed the firm resolution to avoid the same evils. Indeed, I was at once satisfied that, in a majority of cases, the knife, on account of the certainty of profuse and unmanageable hemorrhage, was entirely out of the question.

The ligature of the tumors was not without inconvenience; but it appeared to me that it was defective, not in itself, but in the mode of applying it.

1. The bulk generally enclosed in the ligature was too great to produce a prompt sloughing; and the force required to tighten it sufficiently was productive of the most excruciating pain.

2. In many cases it was impossible to draw the ligature tight enough to occasion the entire death of the parts enclosed in it, without running the risk of cutting partly through the sides of the tumor, contiguous to the ligature: hence, constant pain, irritation, inflammation of the living parts, and fever, were the consequences of the operation. Not unfrequently a second ligature, over the first, became indispensable, before the sloughing was obtained: the parts first detached receded from it, the ulceration was extensive, and the cure protracted to a great length. Yet these evils were trifling, compared to the destruction of the anus, or its contraction so as to require subsequent dilatation, or to leave the patient, for the remainder of his days, with a disease as bad, if not worse, than the former.

3. In cases attended with prolapsus of the gut, the common mode of operating by the ligature is impracticable.

To remedy these defects did not appear a very difficult task. From minor cases to more important ones, I soon satisfied myself that the pain experienced from two or three small ligatures was by no means equal to that given by a single large one; and, consequently was induced to multiply them as much as the bulk of the tumor required.

The only difficulty to be surmounted, was to pass the threads of the ligatures in such a way as to be certain of leaving nothing untied, and to repeat the passage of the needle as seldom as possible, to save pain to the patient; which was obtained by the mode we shall presently explain.

It will be found, as I have long been convinced, that the success and the promptitude of the cure depend, *cæteris paribus*, almost entirely upon the smallness of the parts enclosed in the ligatures, and the certainty of having left nothing out of them.

The mode I have adopted, and successfully pursued for many years, possesses all the advantages aimed at. It differs from others in the following respects:

1. I multiply the ligatures, and enclose in each of them as little as possible of the substance to be removed.

2. I pass two threads with one thrust of the needle, and place the ligatures as close as possible to the sound parts. By this method, one of the threads is used for one ligature, and the other for the next, and not the minutest part can possibly be left out untied; the chain of ligatures resembling the double stitch of the saddler made with two needles.

To perform this operation with ease, and prevent confusion and mistakes, I arm the needle with threads of two different colours. It is then passed through from the side of the anus to the outside of the tumor, and again from this side to the former, and so on alternately until the whole bulk has been stitched all round; observing, between each passage of the needle, to keep a distance of about one-third of an inch, or a little more, according to circumstances, on the outside, and much less relatively on the inside, and leaving behind, after every pull of the needle, a loop of about three or four inches in length.

This part of the operation over, all the loops are to be partly divided as follows:—Cut on the outside all the threads of one colour at the middle of the loops, and all the threads of the other colour next to the anus, in the same manner. You now have evidently a number of isolated ligatures, ready to be pulled and tied closely into a knot. This can be easily and promptly done; and the smaller the portions of the tumor left between each ligature, the less force will be required, and consequently the less pain given to the patient.

All the ligatures being well secured by a double knot, the ends may be clipped off; the patient put to bed; a gentle anodyne given; and a bread and milk poultice put on the parts. It is necessary to observe, that, if the bulk of the tumor operated on be large, it would be well, as the whole of the tumor has now become insensible, to cut away a portion of it, to prevent the fœtor that must eventually result from the decaying substance, particularly during the warm season; and add to every poultice some powdered charcoal. Care, however, must be taken not to cut too close to the ligatures, lest they should slip off, and give rise to troublesome hemorrhage.

In the course of seven to ten days, all the ligatures have dropped: the dressings become very simple, and are to be managed pro re nata.

It may not be improper to state, that I have never seen more, and frequently less, than a teaspoonful of blood lost during the operation, from the largest tumors; and so little fever has supervened, that I do not recollect a single case in which I have been under the necessity of resorting to blood-letting, or recommending a severe diet. Indeed, in the majority of cases, confinement to bed was not rigidly enforced. (*American Med. Recorder.*)

M. Lisfranc's *Mode of Treating Wounds of the Hairy Scalp.*—In wounds of the integuments of the cranium, most authors recommend the immediate reunion of the parts divided, unless the soft parts have been very much bruised and lacerated. This method often succeeds, but it as often happens that the union is only superficial: pus forms beneath, infiltrates the surrounding parts, and produces an inflammation often highly dangerous. M. L. thinks immediate complete reunion should never be attempted in any wound of the hairy scalp: his plan is to shave and carefully clean the circumference of the wound, and, if the edges are much separated, he approximates them by adhesive straps, without, however, bringing them into contact. A space of two or three lines is left between the lips; a little wedge, formed of two or three pieces of lint, is interposed, which should reach quite to the bottom, but not exactly fill it up; for otherwise it would, by compressing the edges of the wound, prevent the flow of pus, and cause the very inconvenience wished to be avoided. A compress with holes in it, covered with cerate, then a piece of lint, and a proper bandage, completes the dressing. If the lips of the wound swell and are painful, the straps should be removed, and the dressing consist of the little wedge compress, covered with an emollient poultice; and this to be continued till every appearance of inflammation is gone. When granulations are formed, the wedge may be discontinued. This plan may be more tedious, but M. Lisfranc considers it much safer, than any other.

I.—Pierre Gindon, aged thirty-two, of good constitution, came into La Pitié, Jan. 11th, with a wound on the top of the head, two inches long, which had quite bared the bone: it had been reunited two days. He had been copiously bled.

12th.—Lips of wound slightly swollen and painful; pulse frequent and full. M. Lisfranc tore open the superficial cicatrix, separated the lips, and allowed some pus to escape. The wound was dressed as recommended above, and a large poultice applied.

14th.—All appearances of inflammation gone; suppuration established; no pain; pulse natural.

22d.—He went away quite cured.

II.—Jean Patten, aged twenty-two, of a sanguineous temperament; February 11th, had a kick from a horse, and was brought to La Pitié. There was a wound of an inch and a half in length, penetrating to the bone, on the right parieto-temporal suture; and another slight wound under the left eyebrow. This was united by adhesive straps; the other was dressed in M. L.'s way. The patient had lost a good deal of blood: he was, however, again bled, and put on low diet.

17th.—Suppuration was established, pain quite gone. The poultice was discontinued, and in a few days he left the hospital quite cured.

Many other such cases might be instanced to show that, by not bringing the lips into immediate contact, subaponeuratic inflammations are avoided. But a surgeon is not always called when the wound is fresh; it may happen that the edges of the wound are already superficially united, and have become the seat of an inflammatory strangulation, more or less intense. What is then to be done? M. L. considers making incisions in such a case very inconvenient, and thinks they should never be had recourse to till other measures have been used, and failed. Indeed, incisions made on inflamed tissues endowed with an exquisite sensibility, cause extreme pain, and make wounds difficult of healing.

Most practitioners employ general bleeding in the cases mentioned. M. Boyer, in his "Traité de Maladies Chirurgicales," says, "The true cause of this inflammation, and the symptoms brought on, is the puncturing or imperfect section of some nervous filament; but a disordered stomach is often added to this, and a bilious disposition. Low diet, general bleeding from the arm and feet, emollient and anodyne fomentations, vomits, when there is derangement of the intestinal canal, laxatives, and chiefly the small doses of the tartrate of antimony, quiet the symptoms and remove the inflammation." It is difficult to imagine that inflammatory strangulation of the wound would disappear under this treatment. Every one knows that general bleedings, which succeed so well in inflammations of parenchymatous organs, as the brain, lungs, &c., is of much less advantage in inflammations of membranous tissues, which are morbidly under the influence of the capillary circulation. Sometimes, when the subject is strong and robust, and symptoms very high, we must have recourse to general before using local bleeding.

When called to a patient under such circumstances, M. Lisfranc tears asunder the lips of the wound, puts a little wedge of lint between them, and covers all with an emollient poultice between two pieces of linen; orders low diet, diluting drinks, sinapisms to the feet; if the intestinal canal is phlogosed, he gives castor-oil, or a mild lavement: but, if the belly be paintul, tongue red, and thirst increased, he prescribes purgatives. It is understood that, if the gastro-enteritis is intense, it must be attacked with leeches to the epigastrium. M. Lisfranc never gives emetics: he thinks they determine too much to the head, in the necessary efforts of vomiting. If the person is strong and full of blood, he prescribes a bleeding from the arm, and immediately after fifteen or twenty leeches, which he allows to bleed for some hours. If inflammation continues next day, and the pulse is not reduced, the leeches are repeated; perhaps even the general bleeding; but the sanguineous depletions must always correspond to the strength of the patient and the violence of the inflammation. As soon as the inflammation is removed, the wound is treated in the ordinary way.

III.—M. Debele, aged sixty-eight, of good constitution, fell, on August 16th, into a cellar, and knocked his head upon the steps in his descent: he fainted, and lost a great deal of blood. He was much bruised, and a wound of more than two inches in length occupied the top of the head. The patient only applied some balsam to the wound.

On the 30th, he came to La Pitié; and, on examination, the lips of the wound were found to be unequally rugged, torn, and much swollen; they were united by a slight cicatrisation. Its circumference had at least four inches of an erysipelatous redness, and was œdematous; the slightest pressure caused excruciating pain, and made him cry out. The face was red and injected; the eyes brilliant; pulse frequent and hard; violent headache.—Venesection; fifteen leeches to each mastoid symphysis. The cicatrix was destroyed, the lips of the wound were kept apart. Ordered a large poultice; low diet, &c.

Next day, symptoms relieved; still inflammatory sloughing of the wound.—Hirudines xxx.

September 2d.—Pain gone; suppuration established;—and, on the 10th, he was dismissed cured.

Many similar cases might be adduced to support M. Lisfranc's treatment. In two cases, the antiphlogistic means, aided by regimen, and the mode of dressing the wound, failed; and incisions were necessarily had recourse to, which relieved the symptoms; but, as always happens, the patient was a long time being cured. (*Revue Medicale.*)

*Wound of the Heart.*—In a late meeting at the Academie Royale de Medicine, M. Ferrus read a case of wound of the heart. A man, thirty years of age, and insane, inflicted on himself a wound, in appearance very trifling, on the left side of the chest, between the fifth and sixth ribs, one inch below and outside the nipple, with a long, thin, and sharp weapon. He was admitted ten days after at the Bicêtre. The wound was then nearly cicatrised, but very painful to the touch; pulse small and intermittent; breathing anxious. Beneath the wound a peculiar noise could be heard,—a sort of undulating crepitation, like that of a varicose aneurism. General bleeding and repeated leeching were made use of; but the respiration became every day more difficult and less free, and the patient died on the twentieth day from the injury.

On opening the body, there was found, at the part of the chest corresponding to the wound, an intimate adhesion of all the internal surface of the left lung to the pericardium: in the cavity of its sac, ten or twelve ounces of a reddish sanies, fetid, and with many discoloured fibrinous clots. The walls of the pericardium were thickened, wrinkled, and evidently inflamed; and a portion of the iron stiletto was planted in the substance of the left ventricle, and firmly wedged in its fibres. It had gone through this ventricle, and penetrated some lines into the cavity of the right ventricle.

M. Ferrus is of opinion that the cause why the man survived so severe an injury for twenty days, was the instrument remaining in the wound, and by its presence acting as a clot in moderating the hemorrhage and effusion of blood into the chest. This, in fact, had taken place gradually, and every day that the man survived it would have been possible to appreciate its progress, the beatings of the heart becoming daily more obscure and deep. It was the effusion that at last killed the patient, the heart not being at all inflamed in consequence of the wound; and that organ becoming accustomed to the presence of a foreign body penetrating it, as in cases of ossifications in the large vessels, the circulation was not, in fact, troubled but for the first days, and at last was quite regular. M. Ferrus thinks that it would have been very dangerous to attempt the extraction of the foreign body, and that the bleedings tended to prolong the patient's life. (*Ibid.*)

*Cases of Extirpation of the Ovarium.*—Dr. A. G. Smith, of Danville, Kentucky, gives the following account of an operation of this kind:—

After putting her upon low diet, and giving aperients and alteratives, for a few days, I performed the operation on the 24th. I commenced by making an incision from the umbilicus to within an inch of the pubis. After cutting down carefully to the peritoneum, I raised it with the tenaculum, and made an incision into it sufficiently large to introduce my finger, and then enlarging it sufficiently to introduce two fingers, I finished the incision by cutting between them. The tumour showed itself immediately, with its peritoneal covering. Finding that it could not be removed through the incision while of the present size, and being unwilling to extend the latter higher up, and believing, too, that the tumour contained a fluid, though deeply seated, I made a large opening into it with the scalpel, and evacuated several pints of watery matter; when it so collapsed, that I was enabled, though not without assistance and difficulty, to get it out of the abdomen. I now found, that the attachment to the right side of the uterus was not much larger than the usual breadth of the broad ligament. The tumour appeared to be an enlargement of the whole ovary, as the fallopian tube, with its fimbriated edge, was stretched over it. I then surrounded the attachment, including the fallopian tube, with a strong ligature of white silk, drew it very tight, and tied it; suffering both ends to remain long enough to extend several inches out of the incision. The tumour was then separated, at least three-quarters of an inch from the ligature. I then turned her over on the abdomen, (at the same time keeping in the intestines with a warm cloth,) to allow all the blood to escape from the cavity; and turning her on her back again, I took five threaded needles, and closed the external incision by the interrupted suture, taking great care to include the peritoneum in the stitches. I left the end of the ligature which included the attachment of the tumour lying out of the lower end of the incision, and secured it by a piece of adhesive plaster; when, after laying on some long pieces of adhesive plaster across the abdomen, and applying a light bandage, I placed her in bed, and gave her seventy-five drops of tinctura opii.

She soon made some efforts to vomit. I then gave fifty drops more laudanum, and, in half an hour after, two hundred per anum, which did not [at] all seem to allay the irritation of the stomach. I then introduced five grains of gum opii, as a suppository; after which, she became easy, and slept an hour. In the evening, I directed a large decoction of senna, salts, jalap, and anise seed, of which she took a wineglassful every two hours, until morning.

(The patient did well; the deep ligature was removed on the twenty-fifth day.)

The first instances of which we are aware, in which this operation was performed by a surgeon, for the removal of a diseased ovarium, are a memorial of the courage of Dr. Ephraim M'Dowell, of Danville, Kentucky. His first case was in December, 1809. The female was here affected with labour-pains; and her case was taken by two physicians for pregnancy. On examination, however, per vaginam, Dr. M'Dowell found nothing in the uterus; and concluded, from consideration, that it was an enlarged ovarium. The incision was made parallel with the rectus abdominis, at the distance of about three inches, and was nine inches long. The tumour was at first too large for extraction. A ligature was put round the fallopian tube, an incision made in the tumour, and about fifteen pounds of a "dirty, gelatinous looking substance extracted. After this, the fallopian tube was cut, and the ovarium extracted, weighing seven pounds and a half. The intestines rushed out of the first opening, and remained out of the abdomen until the tumour was removed. The woman was then laid on her face, to allow the escape of the effused fluids; and the wound afterwards closed with the interrupted suture and adhesive plaster. The ligature of the tube was brought out of the lower end of the wound. In five days after this dreadful operation, the woman was found "engaged in making up her bed;" and, in twenty-five days, she returned home, a distance of sixty miles, in good health.

In the next case, the tumour was immoveably fixed; and was found adhering to the fundus uteri and to the bladder. Having opened the abdomen, however, Dr. M'Dowall made a small incision into the tumour, and removed a large amount of gelatinous matter and blood. He then closed the external wound, and the woman apparently recovered. It is stated, however, in a subsequent communication by the same author, that the swelling gradually returned, and, six years after, became as large as at the period when he operated on her. This patient, a negro woman, was sufficiently relieved, during the interval, to be able to fulfil her usual avocations.

In the third case, the tumour "adhered to the left side." Dr. M'Dowell cut from an inch below the umbilicus to within an inch of the pubis, and then enlarged the opening to the right of the umbilicus, and for two inches above it. He placed a ligature on the tube, as in the first case, and "turned out" an ovarium, which weighed six pounds. No mention is made of the manner in which the adhesions to the left side were overcome. The wound was closed as before. This patient (who was also a negress), recovered in two weeks; excepting the removal of the ligature, which was not effected till after the lapse of five weeks.

In April, 1817, Dr. M'Dowell removed, from another negro woman, a tumour of the ovary, weighing five pounds. The incision was made "near the linea alba." The ligature slipped from the fallopian tube, after its division, this organ being short and spongy; the patient, of consequence, lost a great amount of blood. Ligatures were then applied to several of the arteries individually; but this also failed, as some of them cut through. With much difficulty, a large ligature was now put round the whole stump of the tube, and secured by stitching it in and out of the tube, at several places. This, at last, effected the purpose; and with precautions similar to those in the first case, mentioned above, the wound was closed. The patient recovered of the operation, but remained in ill health; her complaints appearing to be hysterical.

In April, 1818, the same practitioner saw another negro woman, whom he tapped four times, removing at each time a large portion of a gelatinous fluid. He found a firm substance of considerable size, with the probe, which he had introduced to break down the gelatinous masses which presented themselves in the discharge. Finding no marks of pregnancy, per vaginam, he concluded it to be an enlarged ovary. He operated by an incision on the left side. The tumour adhered, by long and slender attachments, to several of the adjacent parts. These adhesions he secured by ligatures, as several of them bled. After a process similar to those described above, the wound was closed. She died, however, on the third day; having been affected with violent pain in the abdomen, and obstinate vomiting. The peritoneum was found violently and extensively inflamed.

Hair and a bone were found in this tumour, as well as a large round perforation, of the size of a musket-ball, opening into the cavity of the abdomen.

These curious cases are described in the Eclectic Repertory, vol. 7, p. 242, and vol. 9, p. 546. It is certainly much to be lamented that the accounts there given of so important an operation are not more copious; and this circumstance has drawn forth comment in the same journal.

In July, 1821, Dr. Nathan Smith, Professor of Physic and Surgery in Yale College, extirpated an ovarium, from a lady of Vermont. It had been observed several years before, when about the size of a goose egg. It burst into the abdomen three times, twice during pregnancy, and once from a fall. At the time of the operation, it exhibited a large tumour in the right side of the abdomen; and was both moveable and capable of internal fluctuation.

The tumour was pushed up into the middle of the abdomen, and an incision, of about three inches long, made in the linea alba, from about an inch below the umbilicus. The peritoneum was not opened till the blood had ceased to flow from the first incision. The tumour was exposed and tapped, and a canula introduced. Eight pints of a dark, ropy fluid were removed. The sac was then drawn out, and separated, with the knife, from the omentum, to which it adhered. Two arteries in the omentum were tied with *leather ligatures.* The ovarian ligament was then divided, two arteries secured as before, and the ligament returned. The adhesion to the parietes of the abdomen was separated by a touch of the knife, and the use of the fingers. The sac was supposed to weigh between two and four ounces. The incision was then closed with adhesive plaster and a bandage.

No unfavourable symptoms occurred. In three weeks the patient was able to sit up and walk; and she afterwards recovered.

Of course, our readers are aware that this operation has been performed successfully by Mr. Lizars, of Edinburgh.

